# Secondary Prevention After Acute Coronary Syndromes in Women: Tailored Management and Cardiac Rehabilitation

**DOI:** 10.3390/jcm14103357

**Published:** 2025-05-12

**Authors:** Luana-Viviana Iorescu, Irina Prisacariu, Chaimae Aboueddahab, Maryam Taheri, Vikash Jaiswal, Ashot Avagimyan, Amine Ghram, Silviu Ionel Dumitrescu, Maciej Banach, Francesco Perone

**Affiliations:** 1 Department of Cardiology, “Dr. Carol Davila” Central Military Emergency University Hospital, 010242 Bucharest, Romania; luanaiorescu@yahoo.com (L.-V.I.); irina.prisacariu96@gmail.com (I.P.); silviudumius@yahoo.com (S.I.D.); 2 Cardiology Department, Ibn Sina University Hospital, Mohammed V University of Rabat, Rabat 10000, Morocco; ch.aboueddahab@gmail.com; 3 Faculty of Medicine, Cardiology Research Center, Hamadan University of Medical Sciences, Hamadan 6517838736, Iran; dr.maryam.taheri@outlook.com; 4 Department of Cardiovascular Research, Larkin Community Hospital, South Miami, FL 33431, USA; vikash29jaxy@gmail.com; 5 Department of Internal Diseases Propedeutics, Yerevan State Medical University After M. Heratsi, Korun Street 2a, Yerevan 0025, Armenia; dr.ashotavagimyan@gmail.com; 6 Cardiac Rehabilitation Department, Heart Hospital, Hamad Medical Corporation, Doha 3050, Qatar; ghram.amine@hotmail.fr; 7 Research Laboratory “Heart Failure, LR12SP09”, Hospital Farhat HACHED of Sousse, Sousse 4031, Tunisia; 8 Ciccarone Center for the Prevention of Cardiovascular Disease, Division of Cardiology, Department of Medicine, Johns Hopkins University School of Medicine, 600 N. Wolfe St, Carnegie 591, Baltimore, MD 21287, USA; maciejbanach77@gmail.com; 9 Department of Preventive Cardiology and Lipidology, Medical University of Lodz (MUL), Rzgowska 281/289, 93-338 Lodz, Poland; 10 Faculty of Medicine, The John Paul II Catholic University of Lublin, 20-950 Lublin, Poland; 11 Cardiac Rehabilitation Unit, Rehabilitation Clinic “Villa delle Magnolie”, 81020 Castel Morrone, Italy

**Keywords:** acute coronary syndromes, cardiac rehabilitation, cardiovascular risk factors, lifestyle management, myocardial infarction, pharmacological treatment, secondary prevention, women

## Abstract

Secondary prevention after acute coronary syndromes is the key strategy to reduce the residual cardiovascular disease risk. A tailored assessment is necessary to suggest the best management and treatment for patients. Sex and gender differences should be strongly considered during cardiovascular evaluation and risk estimation. Indeed, women have a worse outcome than men and are less likely to receive appropriate treatment and evidence-based management. Proper lifestyle management, guideline-directed medical therapy, risk factor management, and cardiac rehabilitation should be recommended early after an acute event in women to reduce the high risk of recurrent events and mortality and improve quality of life. Women-focused cardiac rehabilitation and secondary prevention represent a necessary step in the management and treatment of patients to ensure the best evidence-based care after acute coronary syndromes. This review offers a critical, updated, and comprehensive overview of the appropriate strategies for secondary prevention in women after acute coronary syndromes and long-term treatment, with a focus on cardiac rehabilitation programs. Furthermore, gaps in evidence on this topic and practical recommendations will be provided.

## 1. Introduction

**Figure 1:**
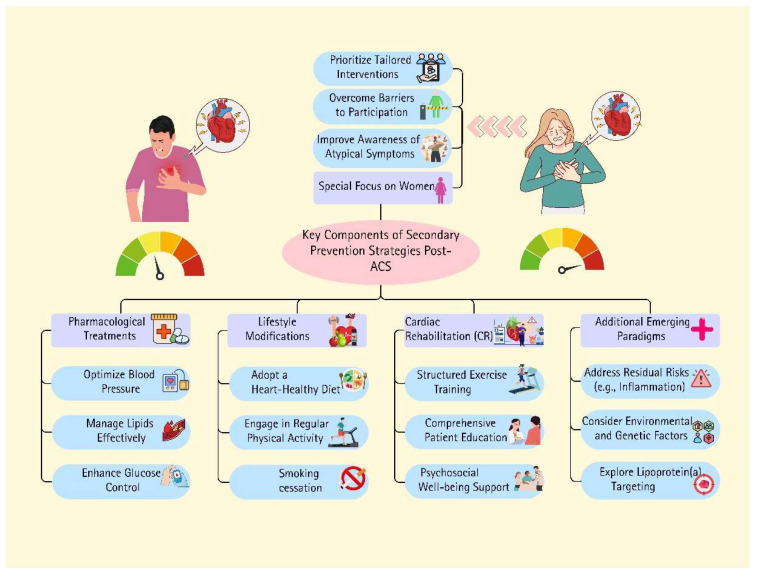
Key components of secondary prevention strategies post-acute coronary syndrome. ACS, acute coronary syndrome.

Cardiovascular diseases (CVDs) remain the leading cause of mortality worldwide, claiming approximately 20 million lives annually. Of these, 85% result from heart attacks and strokes, underscoring the critical contribution of acute coronary syndromes (ACSs) to the overall CVD burden [1]. Patients with a history of ACS face a fivefold higher risk of recurrent cardiovascular events compared to those without known CVD, necessitating robust secondary prevention strategies to mitigate this risk and improve long-term outcomes [2]. Mortality after ACS is also alarmingly high, with as many as 25% of patients dying within 3 years after an event. Secondary prevention following an ACS is a cornerstone of modern cardiovascular care [3]. Its implementation significantly reduces morbidity, mortality, and recurrent cardiovascular events while improving patients’ quality of life [1]. Despite advances in therapeutics, adherence to prescribed treatments and risk factor control remains suboptimal, leaving many patients vulnerable to poor outcomes [4]. Surveys, such as EUROASPIRE V, reveal that a significant proportion of patients fail to achieve recommended targets for blood pressure, lipid levels, and smoking cessation, emphasizing gaps in guideline adherence and risk factor control [5,6]. This highlights the need for a holistic, patient-centered approach that integrates pharmacological treatments, lifestyle modifications, and cardiac rehabilitation (CR) [1,3], as summarized in Figure 1, which provides an overview of the key components of secondary prevention strategies post-ACS.

The post-ACS phase offers a pivotal opportunity to reassess patients’ cardiovascular risk and optimize personalized medical management [1,7,8]. Effective secondary prevention requires not only addressing traditional risk factors such as blood pressure, lipid levels, and glucose control but also acknowledging emerging paradigms like residual cardiovascular and environmental risks [9]. These factors, such as inflammation, lipoprotein(a), and environmental exposures, significantly contribute to adverse outcomes, particularly in high-risk populations [10,11]. Another emerging and independent risk factor is therapy adherence, which may be associated with an increased risk even with the best and most innovative therapies administered [12].

Women face distinct challenges and systemic disparities in ACS management. Their symptoms are often atypical, leading to delayed diagnosis, while lower referral rates for evidence-based interventions, such as CR, contribute to poorer outcomes compared to men [13,14,15,16]. Moreover, women are mostly underrepresented in clinical trials, limiting the applicability of guideline-directed therapies in this population. Addressing these gaps requires specific approaches that consider biological differences, barriers to care, and systemic inequities to ensure equitable and effective treatment [17,18]. CR programs, which combine exercise, education, and psychosocial support, have been shown to significantly reduce morbidity, mortality, and rehospitalization rates while improving medication adherence and quality of life [19]. Despite these benefits, women remain significantly underrepresented in CR programs, with barriers such as referral biases, logistical challenges, and psychosocial factors hindering participation [1]. Participation rates are as low as 11% in women compared to 16% in men, underscoring systemic disparities and the need for tailored interventions to improve accessibility and adherence to CR, which are critical for enhancing outcomes for women after ACS [20]. Therefore, this review aims to provide a comprehensive overview of secondary prevention strategies for women post-ACS, emphasizing tailored management and the integration of CR into care pathways. By synthesizing current evidence, the review will highlight existing gaps, propose practical recommendations for clinical practice, and advocate for specific approaches to optimize long-term outcomes and quality of life.

## 2. Lifestyle Management

Lifestyle management is the foundation of secondary prevention after an ACS in both genders as it involves tailored interventions to optimize recovery, prevent recurrence, and improve long-term outcomes. However, recent evidence suggests that there are notable differences in its implementation and outcomes between women and men due to biological, psychosocial, and behavioral factors. Women, especially post-menopausal women, face unique challenges. Hormonal changes following menopause, including the decline of estrogen, have been linked to an increase in cardiovascular risk.

### 2.1. Tobacco Cessation

For both genders, smoking cessation is a priority after an ACS as it reduces the risk of death by nearly 45%; therefore, behavioral counseling and pharmacological treatment should be used in supporting smokers [1]. Women often face unique challenges in quitting smoking post-ACS compared to men. Research shows that women are more likely to smoke as a coping mechanism for stress, anxiety, or depression, which are more prevalent among women after ACS [21]. Nicotine addiction in women is often influenced by psychosocial factors, such as relationship stress or caregiving responsibilities, making them more susceptible to relapse under emotional strain [22]. Furthermore, women are more likely to experience stronger withdrawal symptoms, including cravings and weight gain, which can discourage sustained cessation efforts [22]. Tailored approaches, such as combining behavioral therapy with nicotine replacement tailored to women’s needs, have been effective in improving cessation rates [21]. Furthermore, electronic cigarettes should be considered, but only with the aim of helping to stop smoking in consideration of the possible harmful impact on health [23,24].

### 2.2. Nutrition and Alcohol Intake

Adopting a heart-healthy diet is another critical aspect of lifestyle management. While women are generally more likely to prioritize dietary changes, such as reducing saturated fats and increasing fruit and vegetable intake, they may face unique challenges. Cultural expectations, food preparation roles, and emotional eating linked to stress can hinder adherence. Men, on the other hand, may struggle with portion control and alcohol consumption. However, weekend alcoholism is more common in women between the ages of 30 and 49. Education on portion sizes and individualized dietary counseling can help both genders achieve sustainable changes [25].

### 2.3. Physical Activity

Extensive data suggest that a sedentary lifestyle is an independent risk factor for all-cause mortality [1]. Engaging in regular physical activity throughout the week, both aerobic and resistance training, is a cornerstone of secondary prevention. However, women are less likely than men to meet recommended physical activity levels post-ACS [26]. Daily step count and mortality are strongly inversely associated, but without significant gender differences [27]. Barriers include caregiving responsibilities, lower self-efficacy, and fear of exacerbating their condition. In contrast, men often perceive exercise as a more achievable and necessary aspect of their recovery. Gender-sensitive interventions, such as group-based exercise programs or home-based CR, have shown promise in increasing adherence among women [28].

### 2.4. Psychosocial Factors

Psychosocial factors play a significant role in lifestyle management as people who have suffered an ACS are reported to have a two-fold risk of mood disorders [1], with women experiencing higher rates of depression and anxiety after ACS compared to men [29]. These conditions can negatively impact motivation and adherence to secondary prevention measures. In men, anger and hostility are more common and may also affect recovery. It is of great importance to identify the patients who need further psychological support and refer them to specialized centers. Integrating mental health support, such as cognitive-behavioral therapy or mindfulness programs, into CR can address these issues effectively [30].

## 3. Risk Factor Management

Significant gender differences exist in risk factor management regarding adherence to treatment targets and access to care, resulting in suboptimal outcomes, particularly for women. Women who experience ACS tend to present with more comorbidities, such as diabetes and hypertension, compared to men, and usually present with atherosclerotic CVD a decade later than men due to the loss of the protective effects of estrogen during the postmenopausal period [31,32]. Moreover, there are also important specific risk factors that contribute to increasing the CVD risk, such as adverse pregnancy outcomes, polycystic ovary syndrome, and premature menopause [32].

### 3.1. Blood Pressure

Managing blood pressure is a cornerstone of secondary prevention after an ACS, as achieving target blood pressure levels significantly reduces the risk of recurrent cardiovascular events. However, gender disparities in blood pressure control and treatment persist, necessitating tailored approaches to management. Women with ACS are more likely to have a history of hypertension and uncontrolled blood pressure compared to men. Despite this, studies suggest women achieve blood pressure targets slightly more often than men, with approximately 45% of women reaching blood pressure goals compared to 38% of men [11]. This difference may be due to women’s higher engagement in healthcare visits and adherence to antihypertensive therapies. However, women are less likely to receive intensive blood pressure-lowering therapy, partly due to concerns about side effects and a lower likelihood of being prescribed combination antihypertensive medications [30,33,34,35].

### 3.2. LDL-Cholesterol

Recent medical literature highlights the importance of aggressive LDL-cholesterol (LDL-C) management in women after ACS, addressing gender-specific differences in risk and treatment response. Women with ACS tend to have worse baseline lipid profiles and a higher prevalence of comorbid conditions, such as diabetes and hypertension, compared to men, which increases their cardiovascular risk [36]. Current guidelines recommend reducing LDL-C to below 55 mg/dL and achieving at least a 50% reduction from baseline to prevent recurrent cardiovascular events [1]. High-intensity statin therapy is the first-line treatment. However, women may experience statin-associated muscle symptoms more frequently (by even 47.9%), leading to challenges in achieving LDL-C goals [36]. When the LDL-C target is not achieved despite maximally tolerated statin therapy, combination therapy with ezetimibe is strongly advised to optimize lipid-lowering outcomes. A PCSK9 targeted therapy is added if this combination also fails, but this therapy is mostly limited, taking into account country-specific reimbursement criteria. Taking into account recent recommendations and availability, bempedoic acid should also be considered. Finally, a new approach to upfront lipid-lowering combination therapy in ACS patients is now also recommended, which allows patients to be on LDL-C goal earlier and lower, with better therapy adherence, lower rates of side effects and discontinuation, and significant reductions in CVOT [1]. Furthermore, new lipid-lowering drugs to lower LDL-C levels should also be considered to reach the goal [37]. Evidence also suggests that women may benefit less from lipid-lowering therapies than men, possibly due to differences in lipoprotein metabolism and underutilization of intensive treatments [36]. Addressing these disparities through personalized, gender-sensitive treatment strategies is critical to improving long-term cardiovascular outcomes for women.

### 3.3. Glycemia

Recent studies underscore the importance of glycemic control in managing risk factors after ACS, particularly in women, who often have distinct challenges compared to men. Recent studies have shown that gender differences in glycemic control after an ACS event may be more significant than previously understood. Women with ACS and diabetes tend to have poorer glycemic control compared to men, which is linked to higher rates of cardiovascular complications and adverse outcomes. One contributing factor is that women are more likely to have higher levels of insulin resistance, which complicates glycemic management [38]. Emerging research also highlights the role of novel diabetes medications in managing glycemic levels in these patients. Sodium-glucose cotransporter-2 (SGLT-2) inhibitors and glucagon-like peptide-1 (GLP-1) receptor agonists are now recommended in treating diabetes among individuals with CVD, with strong evidence supporting their effectiveness in reducing major adverse cardiovascular events (MACEs) and improving glycemic control [39]. Despite these advancements, it remains critical to address the gender-specific factors in glycemic management post-ACS, as women often have more complex and variable outcomes due to factors like weight, hormonal fluctuations, and delayed diagnosis [40].

## 4. Pharmacological Therapy

Women with ACS often face disparities in care compared to men, as demonstrated by their lower likelihood of receiving guideline-directed secondary prevention medications such as dual antiplatelet therapy, lipid-lowering therapy, angiotensin-converting enzyme inhibitors/angiotensin receptor blockers, and beta-blockers (Table 1) [1]. Compounding this issue is the persistent underrepresentation of women in cardiovascular clinical trials [41], which limits the availability of specific data and hampers the development of tailored treatment strategies. This underrepresentation not only undermines the understanding of ACS management among women but also perpetuates disparities in health outcomes.

A large multicenter registry found that, despite women having a higher risk profile, their use of optimal medical therapy (OMT) at 30 days was 5% lower compared to men [42]. This disparity in treatment is attributed to differences in medication initiation rather than treatment adherence. According to the literature, the prescription rates for nearly all guideline-recommended medications were lower in women [43,44,45,46]. In line with this finding, a retrospective population-based cohort study in British Columbia found that lower rates of secondary prevention medication use among women were predominantly observed in younger age groups, driven by differences in treatment initiation. However, in individuals aged from 55 to 64 years, this under-initiation among women was offset by reduced adherence to treatment among men [44]. Multiple trials indicate that women and men exhibit different platelet reactivity and respond differently to antiplatelet therapy. While further research is needed to better understand the complex nature of specific platelet responses, we should adhere to the current guidelines, which recommend identical treatment strategies for both sexes. However, findings from the multicenter Melbourne registry highlight potential inconsistencies in the application of these guidelines. The registry reported that women were significantly less likely than men to receive a second antiplatelet agent. Furthermore, when dual antiplatelet therapy was prescribed, women were more often treated with clopidogrel rather than newer agents such as prasugrel or ticagrelor. Notably, no differences were observed in aspirin prescriptions in this registry [41,42]. The reasons for these disparities remain unclear, but several potential explanations have been proposed. Women presenting with acute myocardial infarction are often older and have a higher baseline risk profile compared to men, which may lead to undertreatment due to concerns about complications. Additionally, female sex is considered a risk factor for bleeding complications following myocardial infarction, as highlighted in the American guidelines [47]. Consequently, women receive antiplatelet agents less frequently due to their higher incidence of adverse bleeding events and vascular complications. However, studies have shown that, after 12 months of dual antiplatelet therapy, there is no significant difference between men and women in terms of major bleeding risk [46,48]. Furthermore, recent meta-analyses have demonstrated no disparities in the efficacy and safety of dual antiplatelet therapy [32]. Adherence to treatment has also been explored between men and women, with some differences identified. A meta-analysis of 28 studies found that adherence to lipid-lowering medication was poorer among women compared to men. In contrast, adherence to antiplatelet therapy, beta-blockers, and renin-angiotensin system inhibitors was similar across genders. This discrepancy may be attributed to differences in drug metabolism between men and women, which could increase the likelihood of adverse effects such as statin-induced myalgia in women, potentially leading them to discontinue treatment [49]. A recent French multicenter nationwide registry, which included patients with acute myocardial infarction from 2005, 2010, and 2015, found that high-intensity lipid-lowering therapy was significantly less prescribed to women. In contrast to findings commonly reported in most studies, women were not associated with poorer prescription of other recommended treatments at discharge, such as beta-blockers or renin-angiotensin blockers [50]. Women were more likely to be prescribed treatments with a less well-established role in current guidelines for secondary prevention after ACS, such as nitrates and calcium channel blockers.

Myocardial infarction with non-obstructive coronary arteries (MINOCA) has a higher prevalence in women than in men. It remains a challenging clinical entity, as there are currently no guideline-directed therapies. Given its heterogeneous nature, identifying the underlying cause is crucial for optimizing treatment strategies. If there is evidence of coronary atherosclerotic disease, risk factor control and secondary prevention therapies should be considered to mitigate future cardiovascular events [1]. Personalized treatment based on the underlying pathophysiology remains key to improving patient outcomes.

A meta-analysis of nine studies examined sex-based differences in outcomes among patients with MINOCA, with aggregate data from five studies specifically assessing medication prescriptions at discharge. The findings indicated that women were less frequently prescribed angiotensin system blockers (ACE inhibitors and ARBs), beta-blockers, and statins compared to men, yet they were more often prescribed aspirin. Despite these disparities in pharmacological management, long-term outcomes, including all-cause mortality, major adverse cardiovascular events (MACEs), stroke, and myocardial infarction, showed no significant differences between the two sexes. This finding may be attributed to the higher prevalence of nonatherosclerotic mechanisms in women with MINOCA [51]. On the other hand, a prospective multicenter study showed different findings, demonstrating that secondary prevention with statins and ACE inhibitors/ARBs was associated with significantly improved outcomes, including reduced mortality, heart failure, and myocardial infarction, in both sexes. Notably, unlike the meta-analysis, this study found no significant difference in the prescription rates of ACE inhibitors/ARBs, beta-blockers, and statins between men and women at discharge. However, a significant sex-based disparity was observed in the use of aspirin, which was prescribed less frequently to women with MINOCA than to men [52]. Beyond the differences between the two sexes, several studies in the literature have examined discharge treatment after MINOCA. In a multicenter registry-based retrospective study, Ciliberti et al. found that the use of beta-blockers was linked to better outcomes, including a reduction in all-cause mortality, heart failure hospitalization, acute myocardial infarction, acute coronary syndrome, and stroke. However, they observed no significant benefit with statins or ACE inhibitors/ARBs [53]. In contrast, Choo et al. reported that ACE inhibitors/ARBs and statins were associated with lower mortality in MINOCA patients. This discrepancy in findings may be due to the limited assessment of the underlying causes of MINOCA in both studies [54]. Further research is needed to better understand the role of different treatments in improving outcomes for these patients [55].

Future research should involve a greater representation of women in clinical trials, alongside increased efforts to explore and better understand gender differences in biology. This could result in more personalized treatments that are better suited to women’s needs.

**Table 1:** Studies on gender disparities in secondary prevention after ACS.

Study	Study Population	Findings
Anand et al. [56] 2005	Patients with ACS, 1998–2000	Women were less likely to be treated with beta-blockers
Jneid et al. [57] 2008	Patients with AMI in 420 US hospitals, 2001–2006	Women were less likely to receive early aspirin treatment and early beta-blocker treatment
Akhter et al. [58] 2009	Patients with ACS, 2004–2006	Women were less likely to receive aspirin or glycoprotein IIb/IIIa inhibitors Women were less often discharged on aspirin or statin
Arora et al. [45] 2019	AMI in four US communities, young patients aged from 35 to 54 years, 1995–2014	Young women were less likely to be prescribed nonaspirin antiplatelet therapy (*p* < 0.0001), lipid-lowering medications (*p* < 0.0001), beta-blockers (*p* = 0.04), and ACEi/ARBs (*p* = 0.02)
Hao et al. [59] 2019	Patients with ACS, 2014–2018	Women were less likely to receive DAPT, renin-angiotensin system inhibitors, and statins at discharge
Vynckier et al. [60] 2020	Patients with coronary heart disease	No gender differences in the prescription of aspirin, beta-blockers, and ACE-I/ARBs Women were less likely on statins at follow-up Women were more likely to receive calcium channel blockers
Dagan et al. [42] 2022	Patients with ACS within the multicentre Melbourne Interventional Group registry, 2005–2017	Women were less likely to receive a second anti-platelet agent (*p* = 0.03), a statin (*p* < 0.001), an ACEi/ARB (*p* < 0.001), and a beta-blocker (*p* < 0.001) compared to men Women were more likely to be prescribed clopidogrel (*p* < 0.001) and less likely to be prescribed ticagrelor (*p* = 0.001)

ACEi, angiotensin-converting enzyme inhibitor; ACS, acute coronary syndrome; AMI, acute myocardial infarction; ARB, angiotensin receptor blocker; DAPT, dual antiplatelet therapy.

## 5. Cardiac Rehabilitation

**Figure 2:**
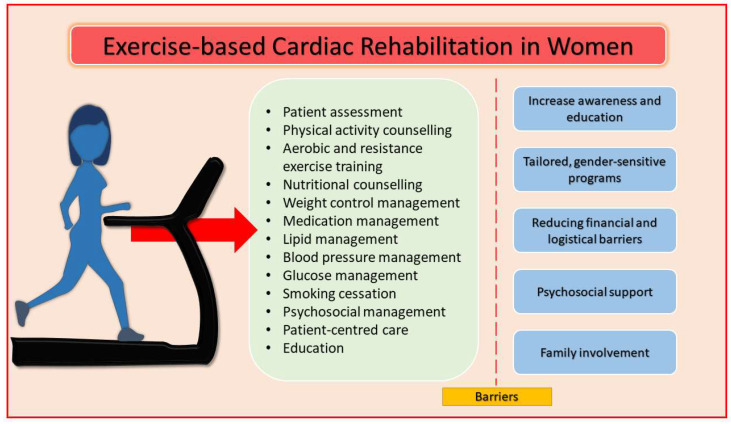
Core components of cardiac rehabilitation in women and strategies to improve participation and adherence.

Exercise training is a cornerstone of CR, helping to improve cardiorespiratory fitness, reduce symptoms, and lower mortality rates after ACS [61,62,63]. For women, supervised exercise is particularly important, especially for those with comorbid conditions, such as diabetes or hypertension, or those who are less physically active. Structured, supervised CR programs are safer, reduce the risk of exercise-induced complications, and enhance patient adherence [64]. Women may also experience different barriers to exercise, making a tailored approach necessary to address individual needs [65]. In women, exercise-based CR includes standard core components consisting of initial assessment, exercise training, physical activity and diet counselling, smoking cessation, and weight, lipid, diabetes, hypertension, and psychosocial management (Figure 2). However, these components present differences between women and men that impact on outcome. Indeed, patient assessment is less accurate in women with reduced cardiopulmonary exercise tests performed during rehabilitation programs [66]. Instead, regarding exercise programs, both aerobic and resistance training are recommended [61,62]. However, women receive suboptimal aerobic exercise training. They show a lower VO2peak both before and after the CR program with less improvement [67,68,69]. Regardless of the impact of aerobic exercise, resistance exercise is associated with a better prognosis in women [70,71]. Regarding physical activity, women engage in exercise less regularly than men. The current guidelines recommend performing at least 150 min of moderate-intensity aerobic exercise per week, or 75 min of vigorous-intensity exercise, combined with strength training two or more days per week [7,24,72].

A comprehensive, multidisciplinary approach to CR is crucial to support the varied needs of women post-ACS. Indeed, secondary prevention includes this key intervention after the acute phase [1]. This collaborative approach ensures that all aspects of recovery (physical, emotional, and nutritional) are addressed, improving overall outcomes and long-term survival [64]. However, although CR is beneficial, several barriers limit women’s access and adherence (Table 2). First of all, women are often under-referred to CR compared to men. Physicians may perceive women as having a better prognosis or be less aware of the substantial benefits CR offers to women [65]. Second, financial constraints, lack of insurance coverage, or difficulty accessing CR centers due to transportation or childcare issues can prevent women from attending [72]. Third, depression, anxiety, and stress are more common in women post-ACS, which can lower motivation to participate in CR. These mental health challenges also affect adherence to prescribed exercise regimens [73]. Fourth, many women may not fully understand the benefits of CR or may feel overwhelmed by the medical and lifestyle changes required. A lack of education about CR can contribute to lower referral rates [72]. Therefore, to improve participation and adherence, several strategies can be implemented:

1. Increase awareness and education: healthcare providers should clearly communicate the benefits of CR, emphasizing how exercise, education, and psychosocial support can reduce the risk of recurrent cardiovascular events and improve long-term quality of life [1].
2. Tailored, gender-sensitive programs: CR programs should account for the unique challenges women face, such as balancing family responsibilities, lower physical fitness, and higher rates of mental health issues. Flexible scheduling, home-based programs, and social support can increase adherence [72,74].
3. Reducing financial and logistical barriers: Programs should offer financial assistance, lower fees, and virtual participation options. Providing transportation support or offering community-based programs can improve accessibility for women [72].
4. Psychosocial support: Addressing psychological concerns is critical. Integrating mental health support within CR programs can help manage stress, anxiety, and depression, thereby improving participation and long-term adherence [72].
5. Family involvement: encouraging family members to participate in CR can provide additional emotional support and increase motivation for women to stick with the program [69].

## 6. Tailored Management for Women After Acute Coronary Syndrome: A Secondary Prevention Strategy

**Figure 3:**
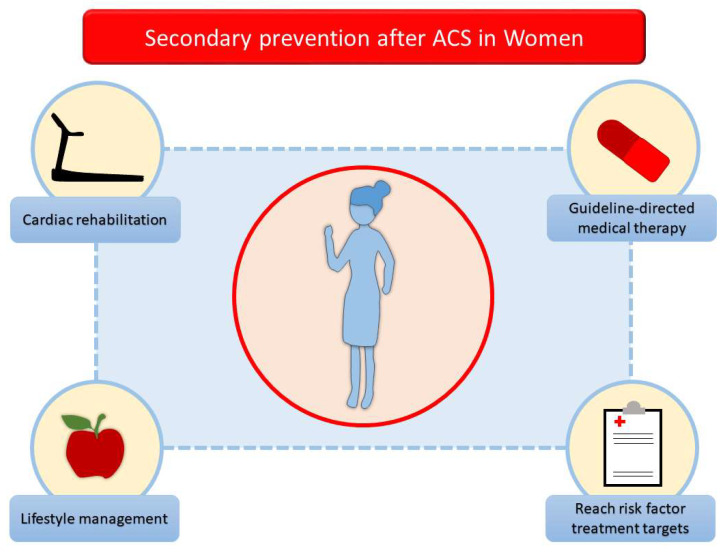
Four pillars of secondary cardiovascular prevention in women after acute coronary syndromes. ACS, acute coronary syndrome.

Management of women after ACS requires a gender-specific approach due to distinct risk factors, clinical presentations, and psychosocial challenges that differ from men. Effective secondary prevention involves optimizing medical therapy, addressing lifestyle factors, promoting CR, reaching risk factor treatment targets, and providing psychological support [1]. A multidisciplinary, personalized strategy is essential to reduce residual risk and recurrent cardiovascular events and improve long-term outcomes for women post-ACS [64]. Indeed, in the acute phase of ACS, immediate interventions aim to stabilize the patient and prevent further cardiac damage [64]. Psychosocial support is crucial during the acute phase, as women are more prone to post-ACS depression and anxiety, which can impede recovery and adherence to treatment. Mental health professionals should be part of the care team to address these concerns [73]. Once the acute phase is managed, the focus shifts to secondary prevention and long-term treatment. This involves pharmacological management, lifestyle changes, risk factor management, and structured CR programs to prevent recurrent cardiovascular events (Figure 3) [72]. These four pillars represent the key strategy to reduce mortality and morbidity and increase the quality of life in women after ACS. Pharmacological treatment should be based on the current ACS guidelines with the aim of reducing the residual cardiovascular risk, improving the prognosis, and continuing the cardio-protective therapy [73]. CR plays a vital role in secondary prevention by improving cardiorespiratory fitness, reducing symptoms, and promoting long-term health. Although women are less likely to be referred to CR and participation rates are lower than for men, tailored CR programs are especially beneficial for women, as they may face challenges like lower physical fitness or deconditioning post-ACS. Psychosocial support integrated into CR programs is also crucial to address anxiety and depression [73]. Mental health issues such as depression, anxiety, and post-traumatic stress disorder are more prevalent in women post-ACS. Addressing these concerns through counseling or support groups is a vital part of secondary prevention. Early intervention can enhance medication adherence, improve participation in CR, and elevate overall quality of life [73]. In this setting, continuous exercise training improves psychosocial distress. Furthermore, women should be advised on lifestyle management to impact their cardiovascular risk and outcome. Smoking is a major modifiable risk factor for recurrent ACS, and support is essential for cessation [72]. Dietary changes, weight management, reduced sedentary time, and regular physical activity are strongly suggested [73]. Women should aim for at least 150 min of moderate-intensity aerobic activity weekly, complemented by strength training. Physical activity should be gradually introduced, particularly for older women or those with comorbidities [1]. Finally, the central goal is to achieve risk factor treatment targets, such as blood pressure < 130/80 mmHg, LDL-C level < 55 mg/dL and reduced ≥50% from baseline, and glycosylated haemoglobin (HbAIc) < 7% in women with diabetes mellitus [1].

## 7. Conclusions

CR is essential for women recovering from ACS, helping improve cardiorespiratory fitness, reduce the risk of future events, and enhance overall well-being. Despite its proven benefits, many women face barriers to referral and adherence to CR programs. A tailored, gender-sensitive approach that addresses specific barriers can improve participation and outcomes for women post-ACS. Also, the management of women post-ACS requires a comprehensive, tailored approach to address their unique needs. This includes early psychosocial support, appropriate medical therapy, achieving risk factor treatment targets, as well as promoting lifestyle modifications and CR. Gender-sensitive strategies will help reduce the risk of recurrent cardiovascular events and improve long-term outcomes for women post-ACS.

## Figures and Tables

**Table 2:** Barriers to cardiac rehabilitation and solutions to improve adherence.

Barrier	Solution
Gender bias in referral	Raise awareness among healthcare providers about the benefits of CR for women
Socioeconomic barriers	Provide financial assistance, sliding scale fees, and transportation support
Psychosocial barriers	Integrate mental health support, counseling, and stress management
Lack of awareness	Educate women about the importance of CR and its long-term benefits
Family responsibilities	Offer flexible schedules and home-based CR options to accommodate caregiving roles

CR, cardiac rehabilitation.

## References

[1] Byrne R.A., Rossello X., Coughlan J.J., Barbato E., Berry C., Chieffo A., Claeys M.J., Dan G.A., Dweck M.R., Galbraith M. (2023). 2023 ESCGuidelines for the management of acute coronary syndromes. Eur. Heart J..

[2] Panattoni G., Monzo L., Gugliotta M., Proietti G., Tatangelo M., Jacomelli I., Zimbardo G., Meringolo F., Fedele E., Calò L. (2023). Optimal management of patients after acute coronary syndrome. Eur. Heart J. Suppl..

[3] Silverio A., Cancro F.P., Esposito L., Bellino M., D’Elia D., Verdoia M., Vassallo M.G., Ciccarelli M., Vecchione C., Galasso G. (2023). Secondary Cardiovascular Prevention after Acute Coronary Syndrome: Emerging Risk Factors and Novel Therapeutic Targets. J. Clin. Med..

[4] Bentzel S., Ljungman C., Hjerpe P., Schiöler L., Manhem K., Boström K.B., Kahan T., Mourtzinis G. (2024). Long-term secondary prevention and outcome following acute coronary syndrome: Real-world results from the Swedish Primary Care Cardiovascular Database. Eur. J. Prev. Cardiol..

[5] Kotseva K., De Backer G., De Bacquer D., Rydén L., Hoes A., Grobbee D., Maggioni A., Marques-Vidal P., Jennings C., Abreu A. (2019). Lifestyle and impact on cardiovascular risk factor control in coronary patients across 27 countries: Results from the European Society of Cardiology ESC-EORP EUROASPIRE V registry. Eur. J. Prev. Cardiol..

[6] Ray K.K., Molemans B., Schoonen W.M., Giovas P., Bray S., Kiru G., Murphy J., Banach M., De Servi S., Gaita D. (2021). EU-Wide Cross-Sectional Observational Study of Lipid-Modifying Therapy Use in Secondary and Primary Care: The DA VINCI study. Eur. J. Prev. Cardiol..

[7] Visseren F.L.J., Mach F., Smulders Y.M., Carballo D., Koskinas K.C., Bäck M., Benetos A., Biffi A., Boavida J.M., Capodanno D. (2021). 2021 ESCGuidelines on cardiovascular disease prevention in clinical practice. Eur. Heart J..

[8] Perone F., Bernardi M., Redheuil A., Mafrica D., Conte E., Spadafora L., Ecarnot F., Tokgozoglu L., Santos-Gallego C.G., Kaiser S.E. (2023). Role of Cardiovascular Imaging in Risk Assessment: Recent Advances, Gaps in Evidence, and Future Directions. J. Clin. Med..

[9] Al-Kindi S., Paneni F., Brook R.D., Rajagopalan S. (2023). Residual environmental risk in patients with cardiovascular disease: An overlooked paradigm. Eur. Heart J..

[10] Gomez-Delgado F., Raya-Cruz M., Katsiki N., Delgado-Lista J., Perez-Martinez P. (2024). Residual cardiovascular risk: When should we treat it?. Eur. J. Intern. Med..

[11] Di Fusco S.A., Arca M., Scicchitano P., Alonzo A., Perone F., Gulizia M.M., Gabrielli D., Oliva F., Imperoli G., Colivicchi F. (2022). Lipoprotein(a): A risk factor for atherosclerosis and an emerging therapeutic target. Heart.

[12] Colantonio L.D., Rosenson R.S., Deng L., Monda K.L., Dai Y., Farkouh M.E., Safford M.M., Philip K., Mues K.E., Muntner P. (2019). Adherence to Statin Therapy Among US Adults Between 2007 and 2014. J. Am. Heart Assoc..

[13] Sheikhy A., Fallahzadeh A., Jameie M., Aein A., Masoudkabir F., Maghsoudi M., Tajdini M., Salarifar M., Jenab Y., Pourhosseini H. (2023). In-hospital and 1-year outcomes of patients without modifiable risk factors presenting with acute coronary syndrome undergoing PCI: A Sex-stratified analysis. Front. Cardiovasc. Med..

[14] Elia E., Bruno F., Crimi G., Wańha W., Leonardi S., Mauro M., Raposeiras Roubin S., Fabris E., Giannino G., Mancone M. (2024). Gender differences in the development of heart failure after acute coronary syndrome: Insight from the CORALYS registry. Int. J. Cardiol..

[15] Holtzman J.N., Kaur G., Hansen B., Bushana N., Gulati M. (2023). Sex differences in the management of atherosclerotic cardiovascular disease. Atherosclerosis.

[16] Sucato V., Comparato F., Ortello A., Galassi A.R. (2024). Myocardical Infarction with Non-Obstructive Coronary Arteries (MINOCA): Pathogenesis, diagnosis and treatment. Curr. Probl. Cardiol..

[17] Dutta D., Mahajan K., Verma L., Gupta G., Sharma M. (2024). Gender differences in the management and outcomes of acute coronary syndrome in indians: A systematic review and meta-analysis. Indian Heart J..

[18] Cader F.A., Sareen N., Press M.C. (2025). Acute Coronary Syndrome in Women. Interv. Cardiol. Clin..

[19] Blacher J., Olié V., Gabet A., Cinaud A., Tuppin P., Iliou M.C., Grave C. (2024). Two-year prognosis and cardiovascular disease prevention after acute coronary syndrome: The role of cardiac rehabilitation-a French nationwide study. Eur. J. Prev. Cardiol..

[20] Norekvål T.M., Bale M., Bedane H.K., Hole T., Ingul C.B., Munkhaugen J. (2024). Cardiac rehabilitation participation within 6 months of discharge in 37 136 myocardial infarction survivors: A nationwide registry study. Eur. J. Prev. Cardiol..

[21] Haider A., Bengs S., Luu J., Osto E., Siller-Matula J.M., Muka T., Gebhard C. (2020). Sex and gender in cardiovascular medicine: Presentation and outcomes of acute coronary syndrome. Eur. Heart J..

[22] Liblik K., Mulvagh S.L., Hindmarch C.C.T., Alavi N., Johri A.M. (2022). Depression and anxiety following acute myocardial infarction in women. Trends Cardiovasc. Med..

[23] Espinoza-Derout J., Shao X.M., Lao C.J., Hasan K.M., Rivera J.C., Jordan M.C., Echeverria V., Roos K.P., Sinha-Hikim A.P., Friedman T.C. (2022). Electronic Cigarette Use and the Risk of Cardiovascular Diseases. Front. Cardiovasc. Med..

[24] Kavousi M., Pisinger C., Barthelemy J.C., De Smedt D., Koskinas K., Marques-Vidal P., Panagiotakos D., Prescott E.B., Tiberi M., Vassiliou V.S. (2021). Electronic cigarettes and health with special focus on cardiovascular effects: Position paper of the European Association of Preventive Cardiology (EAPC). Eur. J. Prev. Cardiol..

[25] Virani S.S., Alonso A., Benjamin E.J., Bittencourt M.S., Callaway C.W., Carson A.P., Chamberlain A.M., Chang A.R., Cheng S., Delling F.N. (2020). Heart Disease and Stroke Statistics-2020 Update: A Report From the American Heart Association. Circulation.

[26] Pelliccia A., Sharma S., Gati S., Bäck M., Börjesson M., Caselli S., Collet J.P., Corrado D., Drezner J.A., Halle M. (2021). 2020 ESCGuidelines on sports cardiology exercise in patients with cardiovascular disease. Eur. Heart J..

[27] Inoue K., Tsugawa Y., Mayeda E.R., Ritz B. (2023). Association of Daily Step Patterns with Mortality in US Adults. JAMA Netw. Open.

[28] Winzer E.B., Woitek F., Linke A. (2018). Physical Activity in the Prevention and Treatment of Coronary Artery Disease. J. Am. Heart Assoc..

[29] Flygare O., Boberg J., Rück C., Hofmann R., Leosdottir M., Mataix-Cols D., de la Cruz L.F., Richman P., Wallert J. (2023). Association of anxiety or depression with risk of recurrent cardiovascular events and death after myocardial infarction: A nationwide registry study. Int. J. Cardiol..

[30] López Ferreruela I., Obón Azuara B., Malo Fumanal S., Rabanaque Hernández M.J., Aguilar-Palacio I. (2024). Gender inequalities in secondary prevention of cardiovascular disease: A scoping review. Int. J. Equity Health.

[31] Rizza V., Tondi L., Patti A.M., Cecchi D., Lombardi M., Perone F., Ambrosetti M., Rizzo M., Cianflone D., Maranta F. (2024). Diabetic cardiomyopathy: Pathophysiology, imaging assessment and therapeutical strategies. Int. J. Cardiol. Cardiovasc. Risk Prev..

[32] Thakkar A., Agarwala A., Michos E.D. (2021). Secondary Prevention of Cardiovascular Disease in Women: Closing the Gap. Eur. Cardiol..

[33] Ramezankhani A., Azizi F., Hadaegh F. (2023). Sex differences in risk factors for coronary heart disease events: A prospective cohort study in Iran. Sci. Rep..

[34] Degli Esposti L., Perrone V., Veronesi C., Gambera M., Nati G., Perone F., Tagliabue P.F., Buda S., Borghi C. (2018). Modifications in drug adherence after switch to fixed-dose combination of perindopril/amlodipine in clinical practice. Results of a large-scale Italian experience. The amlodipine-perindopril in real settings (AMPERES) study. Curr. Med. Res. Opin..

[35] Perrone V., Veronesi C., Gambera M., Nati G., Perone F., Tagliabue P.F., Degli Esposti L., Volpe M. (2019). Treatment with Free Triple Combination Therapy of Atorvastatin, Perindopril, Amlodipine in Hypertensive Patients: A Real-World Population Study in Italy. High. Blood Press. Cardiovasc. Prev..

[36] Alanezi M., Yan A.T., Tan M.K., Bourgeois R., Malek-Marzban P., Beharry R., Alkurtass S., Gyenes G.T., Nadeau P.L., Nwadiaro N. (2024). Optimizing Post-Acute Coronary Syndrome Dyslipidemia Management: Insights from the North American Acute Coronary Syndrome Reflective III. Cardiology.

[37] Di Fusco S.A., Maggioni A.P., Bernelli C., Perone F., De Marzo V., Conte E., Musella F., Uccello G., Luca L., Gabrielli D. (2022). Inclisiran: A New Pharmacological Approach for Hypercholesterolemia. Rev. Cardiovasc. Med..

[38] Lin Z., He J., Yuan S., Song C., Bian X., Yang M., Dou K. (2024). Glycemic control and cardiovascular outcomes in patients with diabetes and coronary artery disease according to triglyceride-glucose index: A large-scale cohort study. Cardiovasc. Diabetol..

[39] Qaseem A., Obley A.J., Shamliyan T., Hicks L.A., Harrod C.S., Balk E.M., Cooney T.G., Cross J.T., Fitterman N., Clinical Guidelines Committee of the American College of Physicians (2024). Newer Pharmacologic Treatments in Adults with Type 2 Diabetes: A Clinical Guideline From the American College of Physicians. Ann. Intern. Med..

[40] American Diabetes Association Professional Practice Committee (2024). 10. Cardiovascular Disease and Risk Management: Standards of Care in Diabetes-2024. Diabetes Care.

[41] Gasecka A., Zimodro J.M., Appelman Y. (2023). Sex differences in antiplatelet therapy: State-of-the art. Platelets.

[42] Dagan M., Dinh D.T., Stehli J., Tan C., Brennan A., Warren J., Ajani A.E., Freeman M., Murphy A., Reid C.M. (2022). Sex disparity in secondary prevention pharmacotherapy and clinical outcomes following acute coronary syndrome. Eur. Heart J. Qual. Care Clin. Outcomes.

[43] Sarma A.A., Braunwald E., Cannon C.P., Guo J., Im K., Antman E.M., Gibson C.M., Newby L.K., Giugliano R.P., Morrow D.A. (2019). Outcomes of Women Compared with Men After Non-ST-Segment Elevation Acute Coronary Syndromes. J. Am. Coll. Cardiol..

[44] Smolina K., Ball L., Humphries K.H., Khan N., Morgan S.G. (2015). Sex Disparities in Post-Acute Myocardial Infarction Pharmacologic Treatment Initiation and Adherence: Problem for Young Women. Circ. Cardiovasc. Qual. Outcomes.

[45] Arora S., Stouffer G.A., Kucharska-Newton A.M., Qamar A., Vaduganathan M., Pandey A., Porterfield D., Blankstein R., Rosamond W.D., Bhatt D.L. (2019). Twenty Year Trends and Sex Differences in Young Adults Hospitalized with Acute Myocardial Infarction. Circulation.

[46] Madonis S.M., Skelding K.A., Roberts M. (2017). Management of acute coronary syndromes: Special considerations in women. Heart.

[47] Mauri L., Smith S.C. (2016). Focused Update on Duration of Dual Antiplatelet Therapy for Patients with Coronary Artery Disease. JAMA Cardiol..

[48] Husted S., James S.K., Bach R.G., Becker R.C., Budaj A., Heras M., Himmelmann A., Horrow J., Katus H.A., Lassila R. (2014). The efficacy of ticagrelor is maintained in women with acute coronary syndromes participating in the prospective randomized PLATelet inhibition patient Outcomes (PLATO) trial. Eur. Heart J..

[49] Bots S.H., Inia J.A., Peters S.A.E. (2021). Medication Adherence After Acute Coronary Syndrome in Women Compared with Men: A Systematic Review and Meta-Analysis. Front. Glob. Womens Health.

[50] Weizman O., Hauguel-Moreau M., Tea V., Albert F., Barragan P., Georges J.L., Delarche N., Kerneis M., Bataille V., Drouet E. (2024). Prognostic impact of high-intensity lipid-lowering therapy under-prescription after acute myocardial infarction in women. Eur. J. Prev. Cardiol..

[51] Ang S.P., Chia J.E., Krittanawong C., Lee K., Iglesias J., Misra K., Mukherjee D. (2024). Sex Differences and Clinical Outcomes in Patients with Myocardial Infarction with Nonobstructive Coronary Arteries: A Meta-Analysis. J. Am. Heart Assoc..

[52] Lawless M., Appelman Y., Beltrame J.F., Navarese E.P., Ratcovich H., Wilkinson C., Kunadian V. (2023). Sex differences in treatment and outcomes amongst myocardial infarction patients presenting with and without obstructive coronary arteries: A prospective multicentre study. Eur. Heart J. Open.

[53] Ciliberti G., Compagnucci P., Urbinati A., Bianco F., Stronati G., Lattanzi S., Dello Russo A., Guerra F. (2021). Myocardial Infarction Without Obstructive Coronary Artery Disease (MINOCA): A Practical Guide for Clinicians. Curr. Probl. Cardiol..

[54] Choo E.H., Chang K., Lee K.Y., Lee D., Kim J.G., Ahn Y., Kim Y.J., Chae S.C., Cho M.C., Kim C.J. (2019). Prognosis and Predictors of Mortality in Patients Suffering Myocardial Infarction with Non-Obstructive Coronary Arteries. J. Am. Heart Assoc..

[55] Nordenskjöld A.M., Agewall S., Atar D., Baron T., Beltrame J., Bergström O., Erlinge D., Gale C.P., López-Pais J., Jernberg T. (2021). Randomized evaluation of beta blocker and ACE-inhibitor/angiotensin receptor blocker treatment in patients with myocardial infarction with non-obstructive coronary arteries (MINOCA-BAT): Rationale and design. Am. Heart J..

[56] Anand S.S., Xie C.C., Mehta S., Franzosi M.G., Joyner C., Chrolavicius S., Fox K.A., Yusuf S., CURE Investigators (2005). Differences in the management and prognosis of women and men who suffer from acute coronary syndromes. J. Am. Coll. Cardiol..

[57] Jneid H., Fonarow G.C., Cannon C.P., Hernandez A.F., Palacios I.F., Maree A.O., Wells Q., Bozkurt B., Labresh K.A., Liang L. (2008). Sex differences in medical care and early death after acute myocardial infarction. Circulation.

[58] Akhter N., Milford-Beland S., Roe M.T., Piana R.N., Kao J., Shroff A. (2009). Gender differences among patients with acute coronary syndromes undergoing percutaneous coronary intervention in the American College of Cardiology-National Cardiovascular Data Registry (ACC-NCDR). Am. Heart J..

[59] Hao Y., Liu J., Liu J., Yang N., Smith SCJr Huo Y., Fonarow G.C., Ge J., Taubert K.A., Morgan L., Zhou M. (2019). Sex Differences in In-Hospital Management and Outcomes of Patients with Acute Coronary Syndrome. Circulation.

[60] Vynckier P., Ferrannini G., Rydén L., Tokgözoğlu L., Bruthans J., Kotseva K., Wood D., De Backer T., Gevaert S., De Bacquer D. (2021). Medical Treatment in Coronary Patients: Is there Still a Gender Gap? Results from European Society of Cardiology EUROASPIRE V Registry. Cardiovasc. Drugs Ther..

[61] Anderson L., Thompson D.R., Oldridge N., Zwisler A.D., Rees K., Martin N., Taylor R.S. (2016). Exercise-based cardiac rehabilitation for coronary heart disease. Cochrane Database Syst. Rev..

[62] Ambrosetti M., Abreu A., Corrà U., Davos C.H., Hansen D., Frederix I., Iliou M.C., Pedretti R.F.E., Schmid J.P., Vigorito C. (2021). Secondary prevention through comprehensive cardiovascular rehabilitation: From knowledge to implementation. 2020 update. A position paper from the Secondary Prevention and Rehabilitation Section of the European Association of Preventive Cardiology. Eur. J. Prev. Cardiol..

[63] Perone F., Spadafora L., Pratesi A., Nicolaio G., Pala B., Franco G., Ruzzolini M., Ambrosetti M. (2024). Obesity and cardiovascular disease: Risk assessment, physical activity, and management of complications. Int. J. Cardiol. Cardiovasc. Risk Prev..

[64] Clark A.M., Hartling L., Vandermeer B., McAlister F.A. (2005). Meta-analysis: Secondary prevention programs for patients with coronary artery disease. Ann. Intern. Med..

[65] Supervia Pola M., Medina-Inojosa J., Yeung C., Lopez-Jimenez F., Squires R., Pérez-Terzic C., Brewer L., Leth S., Thomas R. (2017). Cardiac rehabilitation for women: A systematic review of barriers and solutions. Mayo Clin. Proc..

[66] De Feo S., Tramarin R., Ambrosetti M., Riccio C., Temporelli P.L., Favretto G., Furgi G., Griffo R. (2012). Gender differences in cardiac rehabilitation programs from the Italian survey on cardiac rehabilitation (ISYDE-2008). Int. J. Cardiol..

[67] Rengo J.L., Khadanga S., Savage P.D., Ades P.A. (2020). Response to exercise training during cardiac rehabilitation differs by sex. J. Cardiopulm. Rehabil. Prev..

[68] Savage P.D., Antkowiak M., Ades P.A. (2009). Failure to improve cardiopulmonary fitness in cardiac rehabilitation. J. Cardiopulm. Rehabil. Prev..

[69] De Schutter A., Kachur S., Lavie C.J., Menezes A., Shum K.K., Bangalore S., Arena R., Milani R.V. (2018). Cardiac rehabilitation fitness changes and subsequent survival. Eur. Heart J. Qual. Care Clin. Outcomes.

[70] Saeidifard F., Medina-Inojosa J.R., West C.P., Olson T.P., Somers V.K., Bonikowske A.R., Prokop L.J., Vinciguerra M., Lopez-Jimenez F. (2019). The association of resistance training with mortality: A systematic review and meta-analysis. Eur. J. Prev. Cardiol..

[71] García-Hermoso A., Cavero-Redondo I., Ramírez-Vélez R., Ruiz J.R., Ortega F.B., Lee D.C., Martínez-Vizcaíno V. (2018). Muscular strength as a predictor of all-cause mortality in an apparently healthy population: A systematic review and meta-analysis of data from approximately 2 million men and women. Arch. Phys. Med. Rehabil..

[72] Price J., Landry M., Rolfe D., Delos-Reyes F., Groff L., Sternberg L. (2005). Women’s cardiac rehabilitation: Improving access using principles of women’s health. Can. J. Cardiovasc. Nurs..

[73] Heran B.S., Chen J.M., Ebrahim S., Moxham T., Oldridge N., Rees K., Thompson D.R., Taylor R.S. (2011). Exercise-based cardiac rehabilitation for coronary heart disease. Cochrane Database Syst. Rev..

[74] Angeli F., Ricci F., Moscucci F., Sciomer S., Bucciarelli V., Bianco F., Mattioli A.V., Pizzi C., Gallina S. (2024). Sex- and gender-related disparities in chest pain syndromes: The feminine mystique of chest pain. Curr. Probl. Cardiol..

